# Father absence and pubertal timing in Korean boys and girls

**DOI:** 10.1093/emph/eoad010

**Published:** 2023-05-10

**Authors:** D Susie Lee, Hanna Semenchenko

**Affiliations:** Max Planck Institute for Demographic Research, Konrad-Zuse Strasse 1, 18057 Rostock, Germany; Max Planck Institute for Demographic Research, Konrad-Zuse Strasse 1, 18057 Rostock, Germany

**Keywords:** puberty, father absence, early-life environment, menarche, ejaculation

## Abstract

**Background and objectives:**

Pubertal timing is a key life history trait with long-term health consequences in both sexes. Evolutionary theory has guided extensive research on developmental influences, in particular growing up without a father, on earlier menarche. Far less is known whether a similar relationship exists for boys, especially beyond western contexts. We used longitudinal data from the nationally representative sample of Korean adolescents, which provided us with a unique opportunity for studying male puberty using a hitherto underutilized biomarker: age at first nocturnal ejaculation.

**Methodology:**

We pre-registered and tested a prediction that growing up in father-absent households is associated with earlier puberty in both sexes. Large sample size (>6000) allowed testing the effect of father absence, which remains relatively uncommon in Korea, while adjusting for potential confounders using Cox proportional-hazard models.

**Results:**

Self-reported age at first nocturnal ejaculation was on average 13.8 years, falling within the range known from other societies. Unlike previous findings mostly for white girls, we did not find evidence that Korean girls in father-absent households had a younger age at menarche. Boys in father-absent households reported having their first nocturnal ejaculation 3 months earlier on average, and the difference was evident before age 14.

**Conclusion and implications:**

The association between father absence and pubertal timing appears sex- and age-dependent, and these differences may further interact with cultural norms regarding gender roles. Our study also highlights the utility of the recalled age of first ejaculation for male puberty research, which has lagged in both evolutionary biology and medicine.

## INTRODUCTION

Puberty is a life history transition during which the dominant patterns of energy allocation switch from growth to reproduction [1]. While early reproductive maturation represents an increase of reproductive effort (e.g. earlier first birth [2, 3] or greater reproductive success [4, 5] in females), it is also associated with adverse health outcomes [6] such as risks of sex-steroid-sensitive cancers [7]. Consideration of such life history trade-offs has led research on developmental conditions that mediate adaptive variation in pubertal timing [8]. In industrialized societies, in which energetic stress is less acute, residing in a father-absent household is one developmental condition that has been shown to predict early menarche [9, 10]. Based on the life history theory, it is argued that the earlier initiation of reproductive career is adaptation to the prospect of later adversity, if living without a father either serves as a signal indicative of a harsh environment later in life [11] or directly affects child’s physical conditions that increase later morbidity or mortality [12].

Currently, however, much less is known whether father absence is related to pubertal timing in boys [13–17], reflecting a general lack of research on male life history [18]. This gap in our knowledge is unfortunate, at least for two reasons.

First, although the focus on girls has been explained by higher relevance of trade-offs between growth and reproduction in females [19], human males also face significant costs of reproduction. Humans are among the few mammalian species where paternal care has evolved, especially in the form of direct male care such as carrying young [20]. In humans, paternal care incurs energetic costs [21, 22] involving physiological mechanisms that mediate trade-offs with mating effort [23, 24]. It is thus expected that early puberty, to the extent that it reliably reflects reproductive effort, entails life history trade-offs in males as in females. Indeed, early puberty in boys is a risk factor for adverse health outcomes [6, 25, 26].

Second, sensitivity to early-life environment differs by sex [27, 28]. Evidence suggests that males may be at greater risk of developing diseases as a result of stress experienced during pre-pubertal periods [29, 30]. For example, in a study from Dominica, the stress physiology and testosterone level were more sensitive to presence of father in boys than in girls [31]. Sex differences in the response to father absence may further depend on cultural context, given the distinct developmental contexts created by cultural diversity in gender role and family relationship . As yet, there is insufficient evidence to assess sex differences in the association between family environment and pubertal timing [14] especially beyond western societies [15].

To fill these gaps, the present study used panel data collected in South Korea (hereafter Korea) which provided us with a unique opportunity for studying male puberty using a hitherto underutilized biomarker: age at first nocturnal ejaculation. Male puberty has been understudied in part due to no commonly recognized milestone that is comparable to menarche [32]. The development of secondary sexual characteristics (e.g. pubic hair growth or voice break) is often used as a proxy for pubertal timing in boys. However, changes in these traits occur in stages over time [33], and as such, the exact timing of changes is difficult to pin down especially through self-reports. More importantly, development of secondary sexual characteristics is influenced by adrenarche, which is related to but is independent from gonadarche—growth and maturation of the gonads. Since gonardarche is responsible for menarche in girls and testicular enlargement in boys, biomarkers of gonadarche would reflect the pace of physiological process more directly related to the attainment of fecundity [34–36].

First ejaculation, as a discrete measure of an increase in testicular volume, has been identified as an important milestone in male puberty [37–39]. First ejaculation is closely correlated with bone age [38], and boys tend to provide a clear answer about its timing [39, 40]. Whether self-reported or measured by the presence of sperm in urine, first ejaculation occurs during the stage 3 of sexual maturity rating [41], between 13 and 14 years of age across western and non-western populations [37, 42–45]. Although nocturnal ejaculation occurs as an involuntary physiological reaction during sleep, the experience is conspicuous enough to be remembered especially if it is cultural recognized. In Korea, nocturnal ejaculation is frequently covered in surveys on adolescent health, and even features in TV series and movies.

Based on representative and large sample of Korean adolescents, the present study aimed to (i) describe the distribution of age at first nocturnal ejaculation and age at menarche in Korean adolescents; and (ii) test the prediction that the onset of puberty was earlier for children living in father-absent households. Among studies that tested the prediction in a population of both sexes, findings have been so far mixed [46–50]. We thus started with the prediction for earlier pubertal onset associated with father absence in both sexes. Korea is a high-income country with relatively high social and economic costs associated with divorce particularly for women [51, 52]. As such, although children growing up in father-absent households (usually due to divorce) remain uncommon, not living with father can indicate significant psychosocial stress experienced, as shown among Korean adolescents [53, 54]. From this background, we expected earlier pubertal timing associated with father absence in Korea, similar to findings from western societies.

## METHODOLOGY

We pre-registered the aim and the predictions of this study before conducting the analysis (https://osf.io/d2w9v/). We used R for all data processing and analyses [55].

### Data

**Figure 1.**
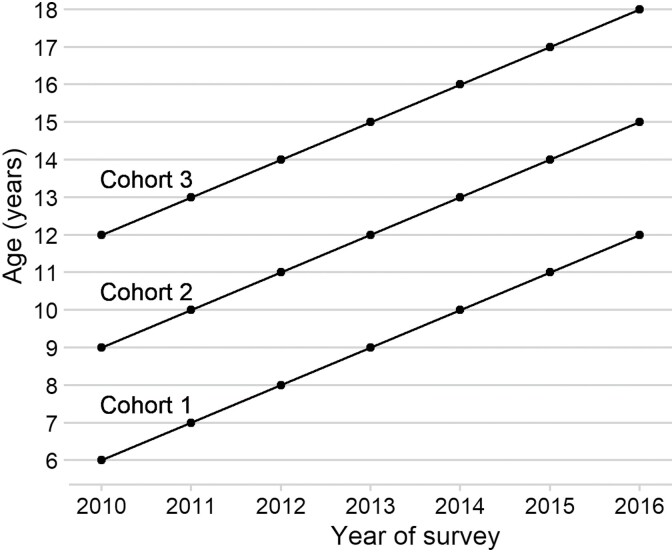
Three cohorts of the KCYPS 2010 data across seven waves. Initial sample sizes were cohort 1 (*n* = 2342), 2 (*n* = 2378) and 3 (*n* =2351). For each of the three cohorts, the proportion of children retained at the end of the survey was 85.5%, 83.2% and 80.0%, respectively.

We used the Korean Children and Youth Panel Survey 2010 data, prospectively collected across seven waves (2010–2016) from 7071 Korean adolescents born around 2000. The youngest, the middle, and the oldest cohorts were 6, 9 and 12 years old, respectively, in 2010 when the survey began (Fig. 1). The survey design necessitated that cohorts cover different age windows: from 6 to 12 for the youngest cohort and from 12 to 18 for the oldest cohort. Since puberty begins mostly at 12 and older, the different age windows meant that the oldest cohort captures the widest distribution of age at pubertal onset, from early starters to late starters.

### Directed acyclic graph

**Figure 2.**
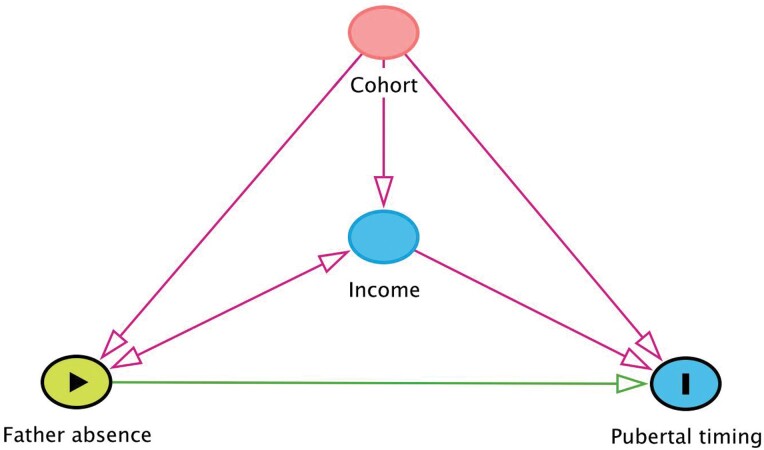
A causal model depicts a postulated relationship between father absence and pubertal timing, as well as potential confounders. The directed acyclic graph was created using the R package ‘dagitty’ [57].

Our causal model (Fig. 2) postulates that father absence affects pubertal timing in both sexes, and the association may be confounded by cohort membership and household income (red arrows leading to father absence and pubertal timing). As cohort differences in the rate of father absence, pubertal timing and household income are expected, the cohort effect was positioned as an overarching confounder. However, we did not expect the father absence and pubertal timing association to differ by cohorts (i.e. interaction), especially given that the studied cohorts are only 3 years apart. For household income, we expect bidirectional relationship with father absence. It is possible that household income predisposes a household for differential risks of father absence (e.g. differential risk of divorce by socioeconomic background), but it is also possible that father absence affects household income (e.g. economic consequences of divorce). Household income can also influence pubertal timing via various routes through which household income has been shown to affect child growth, development and health in Korea [56–58].

### Variables

#### Age at pubertal onset

We used the child’s self-reported age at menarche or first nocturnal ejaculation as a proxy for the age at puberty. For children who reported not having started puberty in the latest survey wave, the observations were censored. For children whose reported ages were inconsistent across different waves (29%), we took the first reported age as the most reliable reminiscence of the pubertal event because memories tend to weaken over time [59]. The degree of inconsistency in reported ages at puberty was small (few months on average; Supplementary Material 1), when the inconsistency was quantified as the difference between the earliest reported age at puberty and the average of inconsistent ages.

#### Father absence

Information about the parental composition in a child’s household over the last year was collected every wave as part of the guardian’s survey (Supplementary Material 2). The proportion of children from single-parents household at the first survey was in average 8%, exactly the same as the national statistic according to the 2018 Single Parent Survey [60].

To process the longitudinal information on parental composition, we took the following steps. First, we selected observations on father absence that were collected before or in the year of the reported age of puberty (or censoring), given that our focus was on the impact of father presence on the timing of puberty. Second, we reclassified nine parental composition values in the original data (Supplementary Material 2) into two states: a child was or was not living with his/her biological father in a given survey year. As a result, a time series of father absence status across years was created for each child. Fourth, to make a variable that captured the ‘average’ father absence status before a child entered puberty (or was censored), we operationalized father absence as ‘not residing with the biological father *most of the time* during the period preceding the onset of puberty’. Lastly, reflecting this definition, a binary variable for father absence was created by assigning a value of one if the proportion of years of father absence in a child’s time series of father absence status was greater than 0.5. The proportion is 0 or 1 for the majority of children whose father absence status was consistent over time. Of note, the proportion of children whose fathers were ‘partially’ present—present and absent during the observed pre-pubertal period—was low (3.54% of the analytic sample; see Supplementary Material 3).

**Figure 3.**
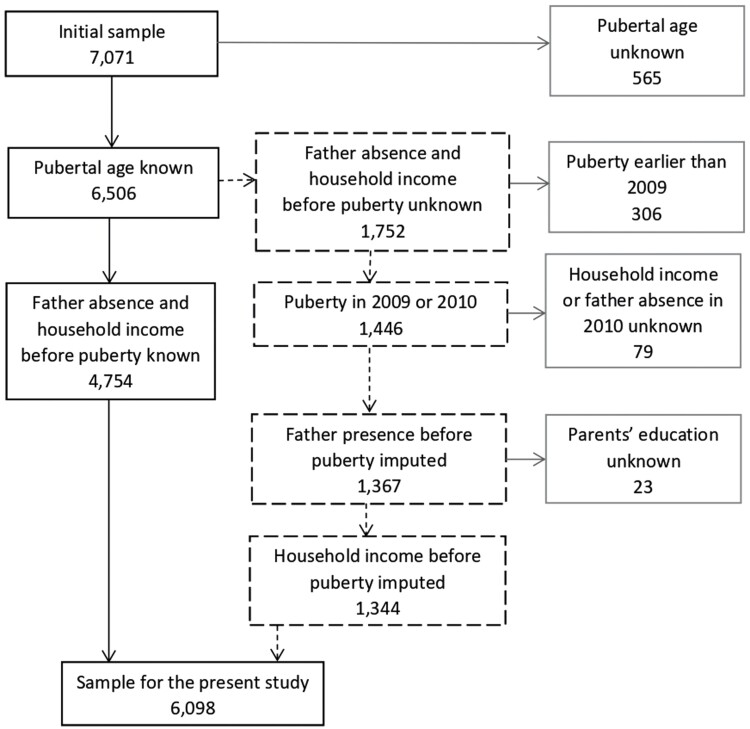
Flowchart showing how the analytic sample (*n* = 6098) was selected from the initial sample (*n* = 7071), and was prepared based on the availability of the necessary information. Excluded samples are indicated in gray-outlined boxes; and samples for which father absence and/or household income had to be imputed are indicated in dash-outlined boxes.

There were 1752 children whose father absence information prior to pubertal onset was unknown (Fig. 3) because their reported age of puberty was before or just in the year of the first survey wave. We were able to impute their father absence status for the majority of these children (*n* = 1367) by relying on the fact that information on 3 years preceding their reported pubertal onset can be inferred from other age- and period-matched KCYPS subjects whose father absence status was observed. However, father absence status of 385 children could not be imputed due to lack of information for imputation, largely among those whose pre-pubertal ages were not covered by the data (69 boys and 237 girls). Most of them were girls, because, as is well known and is also the case in our data, girls begin puberty earlier than boys, and as such, there were more girls—predominantly from the oldest cohort—whose menarche had already occurred in the years not represented by the KCYPS sample (i.e. before the beginning of the survey). Consequently, many girls from the oldest cohort who had menarche at younger age could not be imputed for their pre-pubertal father absence status. Dropping these girls from the analysis did not mean that we have less ‘early starters’, mainly because there were enough early-starting girls from younger cohorts (see Supplementary Material 4.1 for more details).

#### Household income

Questions on annual household income were asked in every wave as part of the guardian’s survey. Because household income was, on average, increasing over time, we used income levels rather than raw income values. Specifically, within each survey year, we assigned raw income values to a three-level ordinal scale—1 = low (below or equal to 50% of median annual income), 2 = middle (above 50% and below or equal to 150% of median annual income), 3 = high (above 150% of median annual income)—based on an annual income distribution indicator available from the Korean Statistical Information Service. For most children (73.7%), the household income level stayed the same across surveys. For cases in which the income level changed over time, we averaged the income levels of each child (e.g. 1-2-2 would be two). For the same reason as for father absence, we imputed the household income for the 1344 children whose pre-puberty information on father absence could be successfully imputed, but whose household income information was missing (supplementary Material 4.2).

### Statistical models

Data from a total of 3237 boys and 2861 girls entered the statistical analyses on the timing of pubertal onset. Using the R package ‘survival’ [61] version 3.2-7, we estimated Cox proportional hazard models separately by sex.

Let $\lambda_{0}\left(t\right)$ be the baseline hazard of entering puberty for children, $X={\left\{X^{F},X^{I}n,X^{C}\right\}}^{⊤}$—vector of covariates such as father absence, income category, and cohort, respectively. Then, λ(t | X)—the hazard of entering puberty given covariates X, assuming they influence baseline hazard proportionally, could be written as follows:


$$\begin{array}{l} \lambda \left(t\;|\;X\right)=\lambda_{0}\left(t\right)\cdot \exp\left(\beta \cdot X\right), \end{array}$$

where $\beta =\left\{\beta^{F},\beta^{In},\beta^{C}\right\}$ is a vector of coefficients to be estimated: father absence, household income, and cohort membership, respectively. For different individuals i and j, relative hazard (hazard ratio)


$$\begin{array}{l} \frac{\lambda \left(t\;|\;X_{i}\right)}{\lambda \left(t\;|\;X_{j}\right)}=\exp\left(\beta \left(X_{i}-X_{j}\right)\right) \end{array}$$

does not depend on time and on baseline hazard. After confirming that the relative hazard of father absence is not constant over time (i.e. the proportionality assumption is not met; Grambsch and Therneau, 1994), we estimated a time-varying coefficient model [62] in which the effect of father absence can change across age. We specified a father absence covariate of the simplest linear type:


$$\begin{array}{l} \beta^{F}\left(t\right)=\beta_{0}^{F}+\beta_{1}^{F}\cdot t \end{array}$$

To check if imputation of father absence and household income make main findings different, we re-estimated the same models based on different subsets of the analytic sample, by excluding the imputed sample or observations from oldest cohort. We also conducted robustness check for the sample excluding cases of partial father absence, and by taking average instead of the earliest reported age in case of inconsistent answers for pubertal onset.

## RESULTS

### Age at menarche or at first nocturnal ejaculation in Korean adolescents

**Figure 4.**
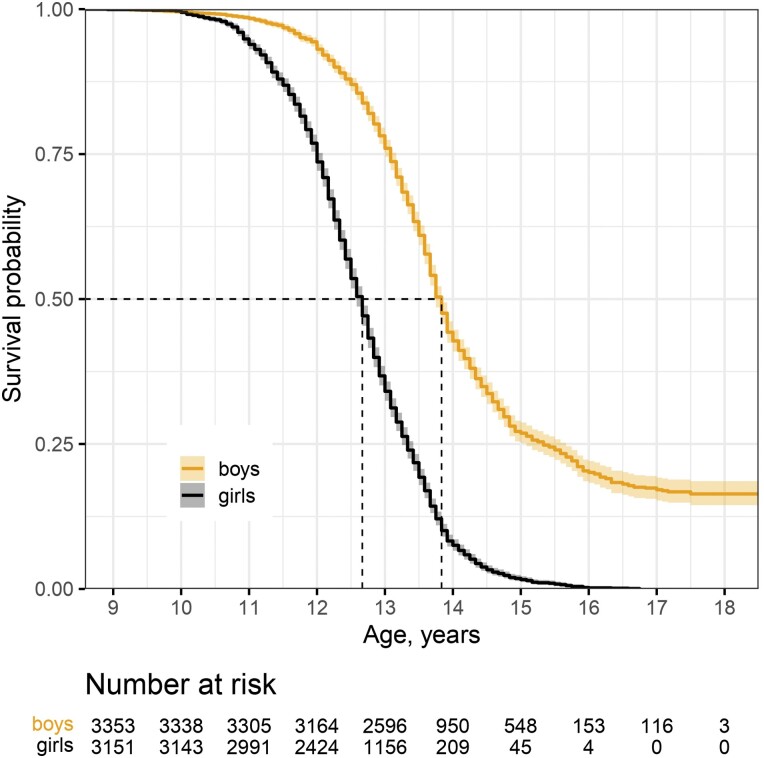
Kaplan–Meier estimates of the survival curves for pubertal age—menarche for girls and first nocturnal ejaculation for boys—in the KCYPS 2010 sample. Note: Calculation based on the KCYPS 2010 sample with known information about the age at puberty or censoring (*n* = 6505).

Age at menarche or at first nocturnal ejaculation was, on average, earlier in girls (12.7 years) than in boys (13.8 years; Table 1, Fig. 4). Likewise, both the earliest and latest reported ages at pubertal onset were earlier in girls 8.9 and 16.8, respectively (9.1 and 17.5 for boys, respectively). While all girls reported experiencing their first menstruation by around age 16, about 16% of boys reported that they had not yet experienced their first nocturnal ejaculation by that age.

**Table 1.** Characteristics of the KCYPS 2010 analytic sample (*n* = 6098), broken down by household income levels

			*Boys* *n* = 3237	*Girls* *n* = 2861
Pubertal age (years)	All		13.76 (±0.02)	12.74 (±0.02)
	By household income	Low	14.08 (±0.25)	12.88 (±0.18)
		Middle	13.83 (±0.04)	12.75 (±0.04)
		High	13.72 (±0.03)	12.74 (±0.02)
Father-absent households^a^ (proportion)	All		4.97%	5.59%
	By household income	Low	19.3%	14.4%
		Middle	59.6%	68.8%
		High	21.1%	16.8%
Father-present households (proportion)	All		95.03%	94.41%
	By household income	Low	1.7%	1.7%
		Middle	26.6%	24.7%
		High	72.7%	74.6%

^a^Proportion of children whose father was absent during most of the pre-pubertal observation period.

### Impact of father absence on the timing of menarche or first nocturnal ejaculation

In our sample (Table 1), 5–6% of children spent the majority of their time prior to the onset of puberty not living with their biological father. Father-absent households were mostly middle-income (64.17%) and similarly distributed between low- and high-income strata, whereas father-present households were predominantly high income (73.58%) and rarely low income (0.69%). There was also an indication of slightly later onset of puberty for children from low-income households, especially in boys.

**Figure 5.**
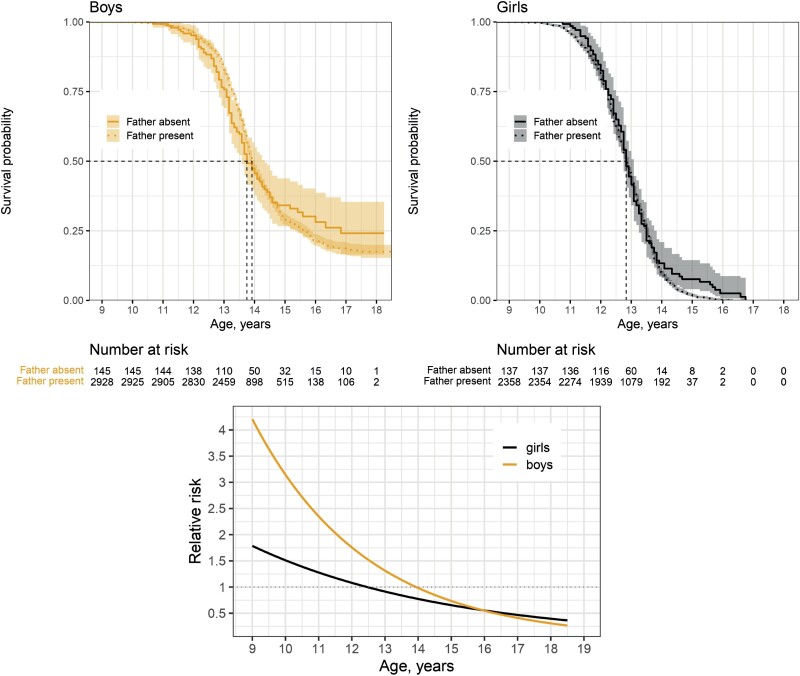
Upper panel: Kaplan–Meier estimates of survival curves for the age at puberty for Korean boys and girls living in father-absent households (solid lines) and in father-present households (dotted lines). Lower panel: Relative risk of pubertal onset for children living without their father compared to that for children living with their father. Calculations of the relative risk are based on the Cox models with father absence as time-varying coefficient (see Supplementary Material 5 for full model outputs.). Note: We performed a calculation using the KCYPS 2010 sample, which included information about age at puberty or censoring, whether a child lived in a father-absent household (most of the time) during pre-pubertal period, and household income (including imputed information, *n* = 6098).

The survival curves of pubertal timing (Fig. 5, upper panel) show that there were differences between boys depending on the father-absence status (log rank test for difference: χ^2^ = 5 on 1 degrees of freedom, *P* = 0.03), but not between girls (χ^2^ = 0.3 on 1 degrees of freedom, *P* = 0.6). Boys who were not living with their father had an average age of first nocturnal ejaculation of 13 years and 2 months, which was approximately 3 months younger on average than boys who were living with their father.

The survival curves further suggested that the impact of father absence on pubertal timing differed by age (Fig. 5, upper panel; see Supplementary Material 5 for model outputs). The Cox models with father absence as a covariate with a time-varying coefficient shed further light on the sex differences in the age-dependency of the impact of father absence (Fig. 5 lower panel). The risk of experiencing nocturnal ejaculation was higher among boys who were not living with their father than among boys who were; however, the effect was evident before age 14, starting from the relative risk of four (95% confidence interval = 2–9) at age nine, and tapering off toward age 14, when the relative risk hit one. At older ages, it was difficult to ascertain the effect due to a lack of statistical power for the small relative risk closer to one. By contrast, the relative risk of pubertal onset among girls living in father-absent households ranged around one at all ages. These findings support the abovementioned observation that there is little difference in average age at menarche by father absence status. The findings remained unchanged when we excluded the sample for which father absence status had to be imputed (Supplementary Materials 6.1, 6.2) or for which the presence of father in a household fluctuated over time (i.e. cases of ‘partial’ father absence; Supplementary Material 6.3), or lastly when we handled inconsistent answers about age at puberty differently by taking averages (Supplementary Material 6.4).

## DISCUSSION

Our results for Korean adolescents suggest that pubertal onset was earlier among boys who were not residing with their father. In previous studies, early first nocturnal ejaculation was associated with the early initiation of sexual intercourse in Korea [45], and was a stronger predictor of sexual behavior than other secondary sexual characteristics in Zimbabwe [42]. Thus, to the extent that early first nocturnal ejaculation predicts the earlier initiation of reproductive effort, our findings for Korean boys provide evidence of faster reproductive maturation among boys growing up in households where fathers are not present most of the time [11, 63]. A crucial next step will be to test whether, and through which pathways, early pubertal onset represents an adaptive response in these boys. For example, early puberty in boys predicts taller height [47, 64], which in turn has been shown to confer greater reproductive success in multiple populations [65, 66].

One possible proximate mechanism by which father absence was related to earlier puberty in Korean boys is the activation of the hypothalamic-pituitary-adrenal axis, which in turn interacts with the hypothalamic-pituitary-gonadal axis [67]. Another possible mechanism is genetic confounding [68] since pubertal timing has a strong genetic basis in both sexes [69]. Although recent works have suggested that father absence is an additive predictor of early menarche [70], similar works are lacking, to the best of our knowledge, to assess the degree of shared genetic basis for father absence and pubertal timing in boys. As such, we cannot rule out genetic confounding as a possible mechanism underlying the observed association between father absence and earlier age at first nocturnal ejaculation.

The overall high gross domestic product per capita, and the government subsidy for one-parent families in Korea, mean that the impact of father absence on children is most likely mediated as psychological rather than energetic or material stress. In our sample, household income was on average lower in father-absent than father-present children, but the low-income households still comprised the minority among father-absent children (Table 1). In a study based on the same KCYPS sample [54], both boys and girls living in single-parent households (which are predominantly single-mother households) reported high overall levels of self-reported stress compared to those living with both parents. However, these findings based on self-reported stress may not reflect the actual stress response, especially given the cultural norms in Korea discouraging the acknowledgement of emotional hardship. Thus, with the lack of physiological data, we cannot yet determine whether the Korean boys who were living in father-absent households indeed experienced heightened stress response prior to pubertal onset, and whether this higher stress response was largely pronounced among boys [31].

In fact, previous studies have shown that marital dissolution—which is the leading cause of nonresidential fathers in high-income countries—may differently affect children by sex. Research mostly from western societies with high divorce rates points at a more negative consequence for boys at least in terms of psychological adjustment [71, 72], and a similar pattern has been reported in a study on Korean school-aged children [73]. These sex differences in early-life effects [27] may in turn interact with social norms about gender roles and their development. For example, in Korean families, strong patriarchal norms centralize male authority running through the father–son axis [74]. In this cultural context, father absence could have a direct impact on the identity of boys as a new patriarch within the household [75], a process that may be amplified by the relatively more negative consequences of divorce on boys as mentioned above. This implies that growing up in a father-absent household may prompt a boy to take on the ‘father’ role at an earlier age, hence earlier reproductive maturation. With no similar cultural expectation for girls, father absence may not have as much significant impact on their reproductive maturation in Korea. Whether this interpretation is held depends on future works from other Asian countries with similar cultural background.

We found little evidence of earlier menarche among Korean girls growing up in father-absent households. This may seem surprising given that the relatively high income and high educational levels in Korea would make the setting similar to other WEIRD (western, educated, industrialized, rich and democratic) societies, where a small but robust association between father absence and earlier menarche has been observed [15]. It is possible that the relatively positive perception of and resilience to parental divorce among Korean girls [73] moderates the stress from living in father-absent households, especially if the strong patriarchal norm renders the meaning of father absence more salient for boys. Other possible moderating factors that may vary by child’s sex, such as types of grandparental support, or quantity and quality of contacts with non-residential fathers, could not be examined in the present study. For example, out of the already small sample of father-absent children (~160 each in boys and girls), less than a quarter of children were living with grandparent(s), making it difficult to examine whether grandparental support matters and differently by sex. Such a low representation of father-absent households, together with the missing pre-pubertal father absence information for more than 200 girls who started menarche before the beginning of the survey (see Methods), may have lowered statistical power for testing the father absence and menarche link, despite the large overall sample size. In this regard, focused sampling of vulnerable groups such as one-parent households holds benefits for future efforts in data collection.

Our findings highlight the utility of the age at first nocturnal ejaculation as a convenient biomarker in the study of male puberty [38, 39, 42, 76]. In higher primates, including in humans, the onset of puberty is initiated when the pulsatile secretion of hypothalamic gonadotropin-releasing hormone (GnRH) resumes after developmental quiescence, and triggers the activation of the pituitary-gonadal axis [34, 35]. In the pubertal transition, GnRH pulses increase particularly at night [77], which may explain why the first spontaneous ejaculation occurs while sleeping. The average age at first nocturnal ejaculation among the boys in our sample was within the range (13–14 years) reported for other societies [39]. It is worth noting that previous studies mostly reported the age at first ejaculation, and not necessarily the age at first *nocturnal* ejaculation, which is broadly understood as an involuntary reaction. Thus, the overlapping age range suggests that the first ejaculation—regardless of how it is achieved—occurs during a specific age window in boys. Moreover, in line with other sex differences in the pubertal transition, the first nocturnal ejaculation was about one year later than the first menarche. For this reason, among children who were followed to age 16.75, all girls reported having experienced their first menstruation, whereas 12.28% boys reported that they had not yet experienced their first nocturnal ejaculation by that age. This observation might also suggest that the first nocturnal ejaculation, unlike menarche, is a milestone that not all boys achieve or recall, or both. More studies across societies with different levels of cultural ‘visibility’ of nocturnal ejaculation could help to clarify these points.

We provide additional evidence that among boys, the impact of father absence is age-dependent. In our study, the impact was restricted to the pubertal transition that occurred during the younger age window of below 14 years. Given that our data on parental composition spanned ages 6 and older, our findings may support the general claim that mid-childhood (juvenility that precedes puberty) is a sensitive period for calibrating sex differences in reproductive strategies [78]. However, without further information from the early childhood or at birth [46], we cannot pinpoint the timing and duration of exposure to father absence and therefore examine the tempo effects on pubertal timing. The KCYPS sample was only asked who were present in the child’s household during the past one year (but not before), and the majority of father-absent children were already not living with their father at the beginning of the survey. Father absence is associated with different family circumstances, ranging from parental divorce, to the death of the father, to temporary separation due to a parent’s job, to co-residence with other family members—each of which is likely to have different effects on a child’s growth and development. According to the 2018 Single Parent Survey [60], most single-parent households in Korea were formed through divorce (77.7%), while smaller shares were formed through the death of a spouse (15.4%), being unmarried (4.0%) or separation (2.9%). Unfortunately, the causes of single parenthood are not further broken down by household type or the age of dependent child even in the Single Parent Survey, to the best of our knowledge. Therefore, we could only assume that majority of the father-absent households in our sample were formed from parental divorce, based on the abovementioned distribution. The lack of detailed family information beyond the absence of the father, as well as the very low proportion of father-absent households in Korea, limited our capacity to interpret the broader implications of our findings.

## CONCLUSION

The pace of reproductive maturation is a key life history trait with crucial implications for population health, even more so given the trends of puberty starting earlier. To help better understand developmental influences on pubertal timing, we fill current gaps in the understanding of male puberty and impact of father absence in non-western societies. We found that Korean boys, but not Korean girls, who did not reside with their father tended to have earlier pubertal onset. Our findings emphasize the need to consider both sexes and across cultures to better understand the effects of early-life conditions, such as father absence, on reproductive maturation.

## Supplementary Material

eoad010_suppl_Supplementary_Material

## Data Availability

The original data can be downloaded from the website of National Youth Policy Institute of Korea (https://www.nypi.re.kr/archive/mps/program/examinDataCode/dataDwloadAgreeView?menuId=MENU00226). The analytic data, which is processed from the original data for the main analyses presented in this manuscript, and R codes used to conduct statistical analyses, are available on the project website (https://osf.io/d2w9v/).
