# Supplementary material for: Father absence and pubertal timing in Korean boys and girls
Source: Evol Med Public Health. 2023 May 10;11(1):174–84. doi: 10.1093/emph/eoad010 (PMC10266580; doi:10.1093/emph/eoad010)
Supplement: eoad010_suppl_Supplementary_Material [file eoad010_suppl_supplementary_material.pdf]

# Supplementary materials for: Father absence and pubertal timing in Korean boys and girls

D. Susie Lee and Hanna Semenchenko

March 23, 2023

## Contents

**1 Inconsistent self-reported ages at puberty** 2

**2 Parental composition types as collected in the original data** 3

**3 Cases of partial father absence in a household** 3
3.1 Description of the cases . . . 3
3.2 Comparison based on different handling of partial father absence . . . 3

**4 Imputation of missing data** 6
4.1 Imputation of father absence . . . 6
4.1.1 Developing a model for father absence . . . 6
4.1.2 Modeling data set . . . 7
4.1.3 Imputing data set . . . 8
4.1.4 Why imputation was not possible for some children and its consequences . . . 8
4.2 Imputing household income . . . 10

**5 Model outputs for the main results** 11
5.1 Boys . . . 11
5.2 Girls . . . 13
5.3 Conclusion . . . 15

**6 Sensitivity analyses** 17
6.1 Excluding the imputed sample . . . 17
6.2 Excluding the oldest cohort . . . 19
6.3 Excluding the cases of partial father absence . . . 21
6.4 Averaging inconsistent answers . . . 23

**Bibliography** 24

## 1 Inconsistent self-reported ages at puberty

Age at menarche or first nocturnal ejaculation were reported by 5169 kids (2148 boys and 3021 girls), 2166 were from oldest cohort, 1824 from middle cohort, and 1179 from the youngest one. 28.6% of them, 1477 altogether (754 boys, 723 girls), gave inconsistent retrospective accounts concerning age at which the event happened.

Due to the survey design, children from the oldest cohort were asked three times about age at puberty, the middle cohort were asked twice, and the youngest cohort were asked only once (likely because the youngest cohort was considered to be too young to have initiated puberty.) As such, not surprisingly, most inconsistent answers on age at puberty come from the oldest cohort, n = 1080 (73%), some from the middle cohort, n = 397 (27%), and none from the youngest. The youngest cohort does not have a possibility of inconsistent answers because they were asked only once.

For those who gave inconsistent answers, we considered two methods for handling such cases. First, we averaged inconsistent ages. Second, we took the first reported age at puberty, i.e., the age that a child provided when s/he gave the first affirmative answer regarding puberty. For example, if a child reported in the first survey to have had menarche at age 12, and then in later surveys gave other ages (10 year in the second and 11 years in the third) as the age at menarche, we took the age reported first – 12 years – as the most reliable one. Even autobiographical memories tend to weaken over time (Bauer and Larkina, 2016), therefore, even for salient events like menarche or first nocturnal ejaculation, recall tends to be more reliable when it is closer in time to the event. So, we preferred and adopted the second method for this study.

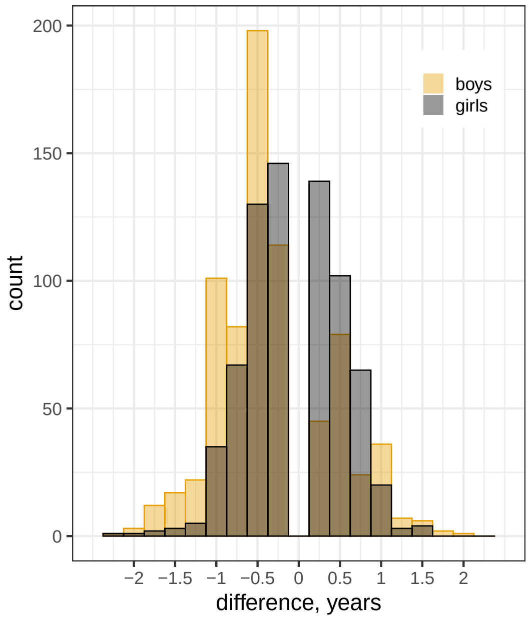


By taking differences between pubertal ages based on the two methods, we sought to understand the degree of discrepancies in the answers regarding pubertal age. Figure on the left shows the distribution of the differences.

The overall discrepancies were small. The distribution of the differences was mostly within the range of -1 and 1. (The distribution is 'empty' at zero, i.e., the cases of consistent answers, because we are only examining children who provided inconsistent answers.) The differences are -0.34 ± 0.03 (mean ± SE), SD = 0.71, and -0.06 ± 0.02, SD = 0.58 for boys and girls, respectively. The differences are smaller in girls, presumably because of the higher salience of menarche than first nocturnal ejaculation.

In the section 6.4 of this document, we present results from a sensitivity analysis based on the sample for which pubertal age was calculated alternatively by taking averages for inconsistent answers. Sensitivity analyses on the sample excluding the oldest cohort is presented in section 6.2.

We did not consider removing records with inconsistent reports since our goal was to find a way to include cases with inconsistencies rather than exclude them from the sample. It is important to retain them, especially considering that the distribution of inconsistent answers largely stems from the survey design rather than characteristics of children prone to inconsistent recall. Our approach for this study treats all cohorts equally. Therefore, from a statistical perspective, our approach is preferrable to excluding a disproportionate number of cases from different cohorts.

## 2 Parental composition types as collected in the original data

Nine types of information on the parental composition of child's household were collected. The values 1, 2, 4 indicate the information we coded as 'father present' status.

| Value for the parental composition variable | Meaning |
|---|---|
| 1 | both biological parents are present |
| 2 | only biological father |
| 3 | only biological mother |
| 4 | biological father and stepmother |
| 5 | biological mother and stepfather |
| 6 | stepfather and stepmother |
| 7 | only stepfather |
| 8 | only stepmother |
| 9 | any parents are absent |

## 3 Cases of partial father absence in a household

### 3.1 Description of the cases

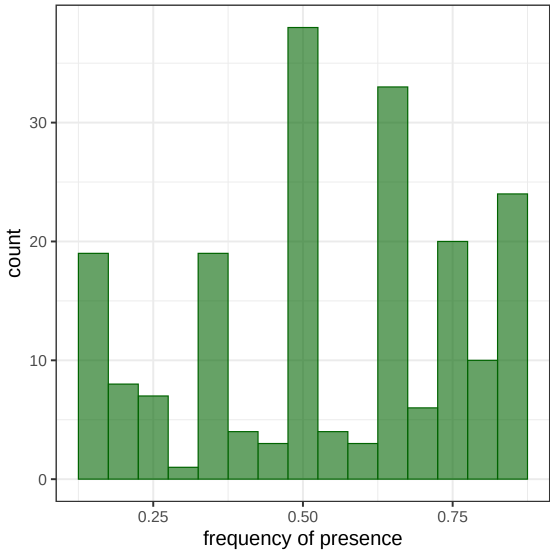


Within the analytic sample, there were 234 (4.16%) and 5186 (92.29%) cases in which a biological father was consistently absent or present, respectively, during the pre-pubertal time period that was observed in the data.
A biological father was observed to be 'partially' absent (i.e., present and absent over time) among 199 (3.54%).
The histogram on the left displays the fraction of time father was present in the household before child's puberty (or censoring) among those cases of partial father absence.

### 3.2 Comparison based on different handling of partial father absence

In this section, we considered different ways of handling the cases of partial father absence. The goal was to see if the differences in pubertal timing by father absence were robust to different specifications of partial father absence.

For each specification of partial father absence, we compared Kaplan-Meier (KM) curves for pubertal timing by father absence. References to the respective KM estimators of the survival curves, tables with number at risk for different ages, and $\chi^2$ statistics are given in parenthesis. Subsample includes only those for whom information on family composition is available before and/or in the year of the reported age at puberty.

1. Three categories of father presence: absent, present all the time, partially present (Fig. 1). That is, the cases of partial father absence are considered as a separate group.

2. Father was absent, if he was reported absent at least once before puberty (Fig. 2).

3. Father was present, if he was reported partially present before puberty (Fig. 3).

4. Father present/absent all the observable time before puberty. That is, cases of partial father absence are removed. (Fig. 4).

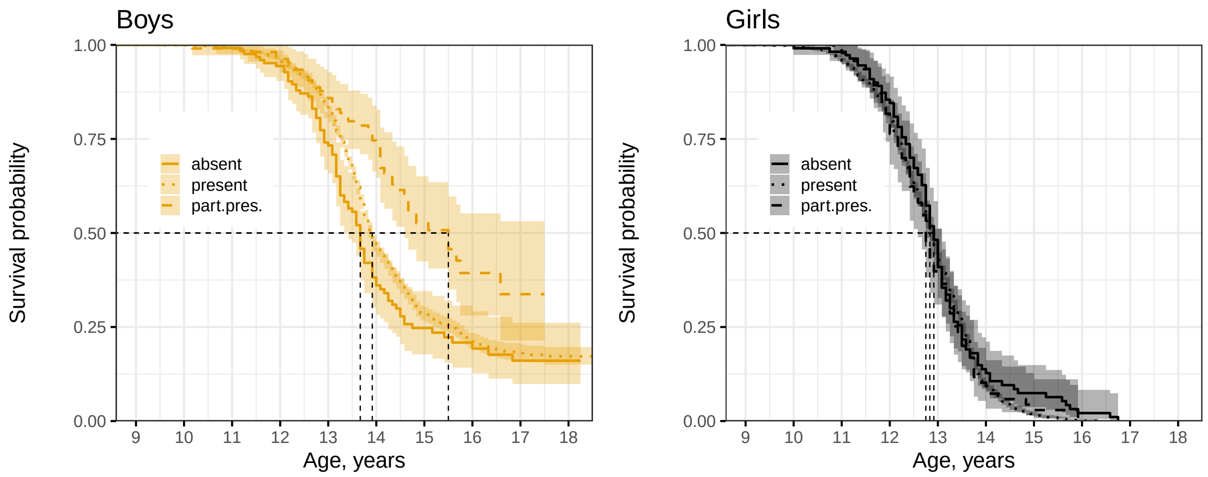


Number at risk (Boys)

| | 9 | 10 | 11 | 12 | 13 | 14 | 15 | 16 | 17 | 18 |
|---|---|---|---|---|---|---|---|---|---|---|
| absent | 124 | 124 | 123 | 117 | 91 | 37 | 22 | 13 | 8 | 1 |
| present | 2872 | 2869 | 2850 | 2775 | 2412 | 862 | 495 | 133 | 102 | 2 |
| part.pres. | 106 | 106 | 105 | 103 | 92 | 51 | 31 | 7 | 6 | 0 |

Number at risk (Girls)

| | 9 | 10 | 11 | 12 | 13 | 14 | 15 | 16 | 17 | 18 |
|---|---|---|---|---|---|---|---|---|---|---|
| absent | 110 | 110 | 108 | 94 | 53 | 13 | 7 | 2 | 0 | 0 |
| present | 2313 | 2309 | 2231 | 1905 | 1066 | 188 | 35 | 2 | 0 | 0 |
| part.pres. | 93 | 93 | 92 | 76 | 36 | 7 | 3 | 0 | 0 | 0 |

Figure 1: The KM estimators of the life before puberty duration for boys and girls with and without father, and with father partially present. (boys: $\chi^2 = 24.2$ df= 2, p= 6e-06; girls: $\chi^2 = 0.8$ df= 2, p= 0.7)

According to the first definition, partial father absence was categorized as a separate group, such that pubertal timing was compared between three groups: "father absent", "father present" and "father partially present". Fig. 1 shows that there is no difference in age at menarche by father absence. For boys, we continue to see earlier age at first nocturnal ejaculation among those whose father was absent. It is interesting to see delayed age at first nocturnal ejaculation among boys from a household where father was only partially absent during the pre-pubertal time. Available data do not allow for hypothesizing and testing possible mechanisms. One possibility is that late puberty and thus a long observation window for pre-pubertal period makes it more likely that the status of father absence varies over time. (That is, longer an observation period, higher chance that longitudinal observations fluctuate).

As for the second definition, which lumps all partial father absence as 'father absence', the difference in empirical survivals is not significant for both male and female respondents (Fig. 2). On the contrary, if all partial father absence is lumped as 'father presence', the empirical survivals demonstrate acceleration of puberty among boys from households without father, compared to those whose father was present or partially present during pre-pubertal time (Fig. 3). This is well expected based on what we have already observed from Fig. 1 which was based on the first definition.

We decided to reject both second and third definitions on the ground that they artificially give more weight to father absence/presence, and increase/decrease the numbers of fatherless households, respectively. Moreover, those whom were observed longer has more chances to report father absence at least once. Attributing partial father presence to either category results in elevation of the tail of corresponding survival curve.

Lastly, according to the fourth definition, we removed the cases of partial father presence. In other words, we reduce our sample to only those, whose father's absence/presence could not be questioned. In this case, significant difference is observed among boys, father absence is associated with accelerated puberty (Fig. 4). There is no difference in girls' survival curves. Given that there are not many cases of partial father absence, it is perhaps not surprising that the results do not differ from our main findings.

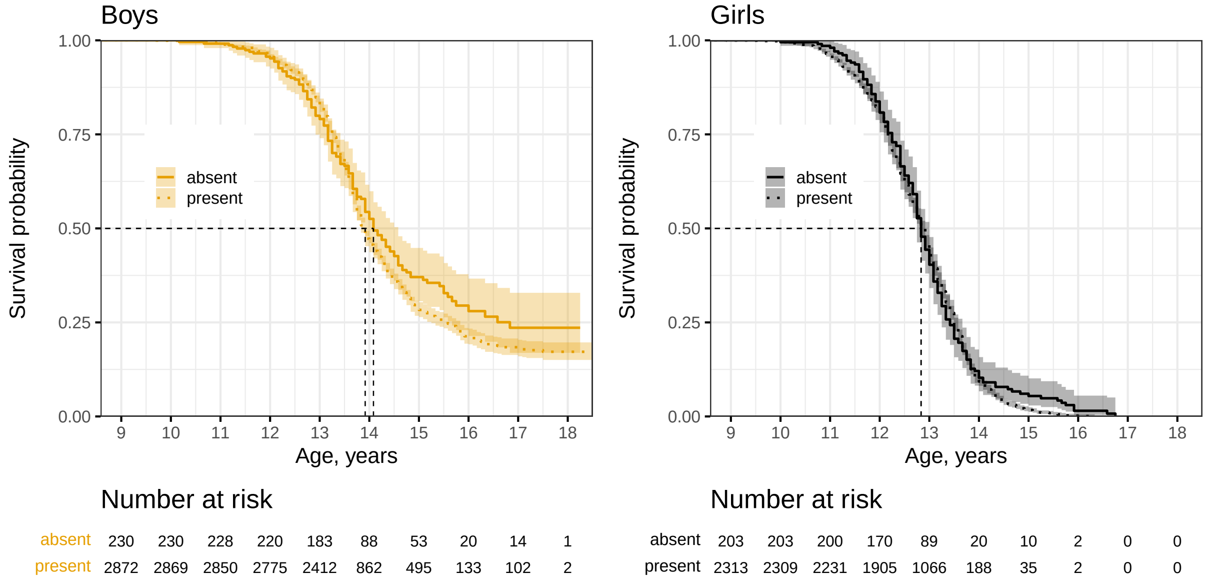


Figure 2: The KM estimators of the life before puberty duration for boys and girls with and without father at least once. (boys: $\chi^2 = 0.7$ df= 1, p= 0.4; girls: $\chi^2 = 0$ df= 1, p= 0.9)

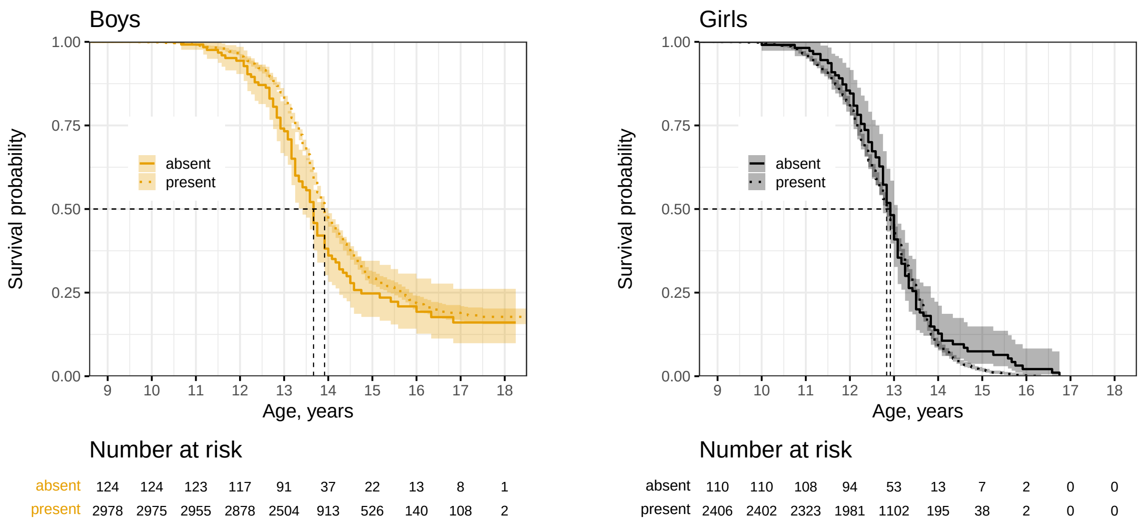


Figure 3: The KM estimators of the life before puberty duration for boys and girls with and without father, partially presence treated as presence. (boys: $\chi^2 = 8.8$ df= 1, p= 0.003; girls: $\chi^2 = 0.5$ df= 1, p= 0.5)

In the section 6.3 of this document, we present results from a sensitivity analysis based on the sample excluding partial father absence.

From considering different ways of handling the cases of partial father absence, we conclude that the approach we take for the main analyses — i.e., assigning father absence if more than 0.5 of observed pre-pubertal period was father-absent — is preferrable, because it allows for utilization of bigger sample, distributes the group with partially present father between the two categories without attributing unjustified weight to either, and, at the same time, ensures that cases of late puberty with long observation period are present in both marginal groups.

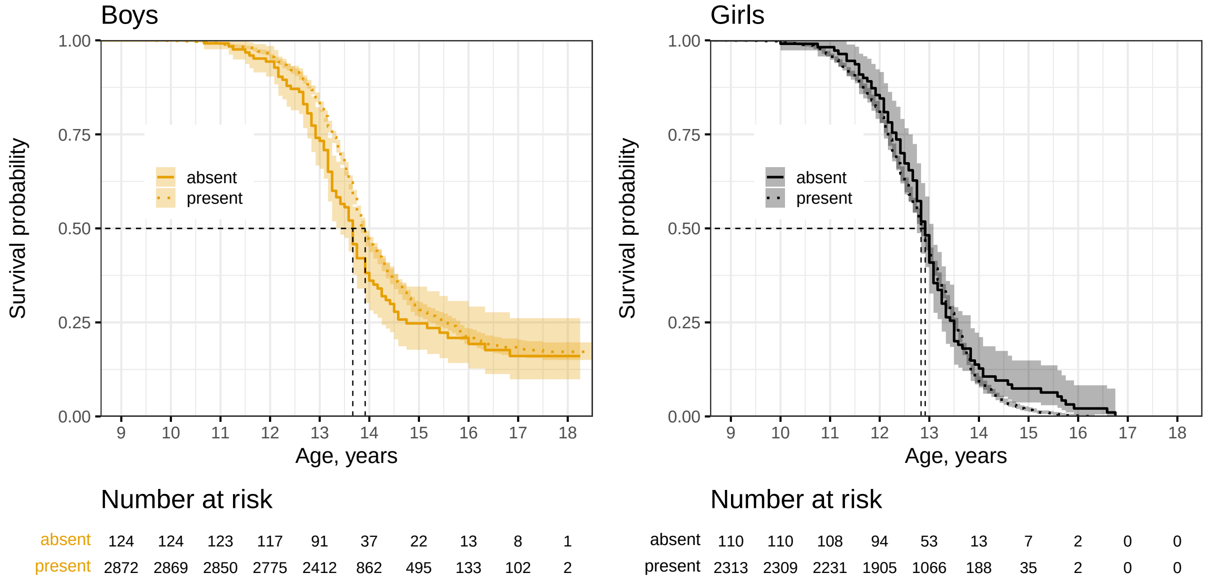


Figure 4: The KM estimators of the life before puberty duration for boys and girls with and without father all the time during the observation. (boys: $\chi^2$ = 7.9 df= 1, p= 0.005; girls: $\chi^2$ = 0.4 df= 1, p= 0.5)

# 4 Imputation of missing data

## 4.1 Imputation of father absence

We imputed father absence status for whom (n = 1,367) we could make use of the fact that children from younger cohorts were observed across the ages equivalent to the 'pre-puberty' period of the children whose pre-puberty parental composition is unknown. We started from the question: What is the probability that a child spent the majority of time without father during the preceding 3 years, given the information whether they were living with or without a father at the age of the 1st grade of middle school and child's background (sex, region of residence, household income)? To answer this question, we took a two-step approach. We first developed a model for father absence, and then used an obtained model to impute father absence status for the 1,367 children.

### 4.1.1 Developing a model for father absence

Model: We used logistic regression to model the probability of father presence for each child during age interval of interest, which corresponds to three years before the 1st grade of middle school. Let $p$ be the probability of father presence in a given age interval, then its log odds could be represented as linear combination of covariates $x_i$ (sex, region of residence, household income):

$$\ln\left(\frac{p}{1-p}\right) = \beta_0 + \sum_{i=1}^{n} \beta_i x_i,$$

where $\beta_i$, $i = 1, ..., n$ are parameters to be estimated. When values of $\beta_i$ are estimated, probability $p$ is obtained from the reciprocal of logistic function:

$$p = \frac{1}{1 + e^{-\left(\beta_0 + \sum_{i=1}^{n} \beta_i x_i\right)}},$$

Then, following our operationalization of father presence given changes in father presence status across time (see main text), we assign 'father absence' if $p < 0.5$ and 'father presence' if $p \geq 0.5$.

We used the binomial family of generalized linear model with `logit` link function, i.e. `glm()` function in R.

Variables: To predict the probability of father presence three years earlier, we use information on child's sex, region of residence, household income and father presence at the time of the 1st year of middle school. The 1st year of middle school is the youngest age at which the father absence status of the children for whom we had to impute father absence were known. Our goal is to impute father absence status preceding this age, `father_before`, using the information from children whose father absence was known at and before the 1st year of middle school.

We prepared the predictor variables as follows. For sex `gen`, we recoded the `gender` variable from the original data set into 1 and 2 for boys and girls, respectively. For region `reg`, we used the variable `ara2awN` from the original data set. It takes 17 values for different highest-level subjects of administrative division of South Korea. This division consists of 1 special city (Seoul), 1 special self-governing city, 6 metropolitan cities and 9 provinces including a special self-governing province. Income increased over the survey period (2010-2016). To remove the time trend in the income data, we considered two approaches and introduced two income variables, `inc_q` and `inc_st`, based on `incomewN` variable from the original dataset, where `N` is the wave number. `inc_q` is raw income categorized as quartiles of the income distribution, and `inc_st` is scaled or standardized income variable with 0 mean and standard deviation of 1. Both transformations were applied to raw income within every cohort for selected years using all available information on individual household income. Lastly, for the father absence status on the 1st year of middle school, `father_now`, we used information on the father presence/absence for respective years, recoded from parental composition given in original data.

All variables described above served as covariates in regression either separately or in some combination.

#### 4.1.2 Modeling data set

Data for developing a model on father absence: We selected a subsample of children (n = 4009) who have information on all the above-mentioned variables.

Comparison of models: To test predictive performance of different models, we randomly divided the modeling data set into `train` and `test` subsets (containing 65% and 35% of data, respectively). On `train` subset we estimated the parameters of different models and then tested their predictive abilities on `test` subset. We compared fitted models by residual deviance (ANOVA was performed on the nested models) and Akaike information criterion (AIC).

Comparison of models using ANOVA shows that variables `father_now`, `inc_q` and `reg` describe the data better than the model with two covariates.

If standardized values of income are used, the three covariates `father_now`, `inc_st` and `reg` can predict `father_before` better than all mentioned above.

It turned out that the sex of a child does not improve significantly the fit the model to the data.

Table 1: Analysis of Deviance Table

| | | Df | Deviance | Resid. Df | Resid. Dev | Pr(> $\chi$) |
|---|---|---|---|---|---|---|
| | NULL | | | 2599 | 923.53 | |
| m1 | `father_now` | 1 | 620.26 | 2598 | 303.27 | <2.2e-16*** |
| m2=m1+ | `reg` | 15 | 20.57 | 2583 | 282.70 | 0.151230 |
| m3=m2+ | `inc_q` | 1 | 14.52 | 2582 | 268.19 | 0.000139 *** |
| m4=m3+ | `gen` | 1 | 1.03 | 2581 | 267.15 | 0.309004 |
| m5=m2+ | `inc_st` | 1 | 21.69 | 2582 | 261.01 | 3.202e-066*** |
| m6=m3+ | `gen` | 1 | 1.27 | 2581 | 259.74 | 0.2591 |

Signif. codes: *** 0.001, ** 0.01, * 0.05, · 0.1

Based on these comparison results, we chose a model with three covariates, `father_now`, `inc_st` and `reg`, for data imputation. Prediction of father presence in `test` subset shows that the model m5 produces

Table 2: Coefficients of logistic model with log-odds of father presence as a response

| Model | AIC |
|---|---|
| m3 | 304.19 |
| m4 | 305.15 |
| m5 | 297.01 |
| m6 | 297.74 |

18% false positive and 92% of true negative results (m6 is the same). Models m3 and m4 predict smaller number of false positive (15%) and the same number of true negative cases. According to the AIC m5 describes data better than all others, so, this is the model of our choice. The estimated parameter values for this model are given in the table 3.

Table 3: Coefficients of logistic model with log-odds of father presence as a response

| | Estimate | Std. Error | z value | Pr(> $\|z\|$) | |
|---|---|---|---|---|---|
| (Intercept) | 0.9916 | 0.6574 | 1.508 | 0.131480 | |
| father_now1 | 6.3308 | 0.4469 | 14.166 | <2e-16 | *** |
| inc | 1.1421 | 0.3152 | 3.623 | 0.000291 | *** |
| reg20 | -2.7562 | 0.8919 | -3.090 | 0.002000 | ** |
| reg21 | -1.1694 | 0.9899 | -1.181 | 0.237498 | |
| reg22 | -1.6727 | 1.0249 | -1.632 | 0.102655 | |
| reg23 | -0.6965 | 1.7324 | -0.402 | 0.687651 | |
| reg24 | -2.0874 | 1.0981 | -1.901 | 0.057309 | · |
| reg25 | -2.2692 | 1.0555 | -2.150 | 0.031561 | * |
| reg30 | -0.9055 | 0.7543 | -1.201 | 0.229943 | |
| reg31 | -2.2309 | 1.0142 | -2.200 | 0.027837 | * |
| reg32 | -0.4681 | 0.9675 | -0.484 | 0.628492 | |
| reg33 | -2.7566 | 0.8982 | -3.069 | 0.002148 | ** |
| reg34 | -2.4151 | 1.0134 | -2.383 | 0.017167 | * |
| reg35 | -2.4348 | 1.0236 | -2.379 | 0.017372 | * |
| reg36 | -1.6090 | 1.0394 | -1.548 | 0.121627 | |
| reg37 | -1.8973 | 0.9610 | -1.974 | 0.048357 | * |
| reg38 | -0.4775 | 0.9557 | -0.500 | 0.617345 | |

Signif. codes: *** 0.001, ** 0.01, * 0.05, · 0.1

#### 4.1.3 Imputing data set

We have 1,752 children who started pubertal changes before the beginning of the survey, i.e. in 2010 or earlier, and as such, no information about parental composition available before the onset of puberty. Among them, 1,446 children whose reported age of puberty was in 2009 or 2010. For 79 of them, there was no information on income and father presence in 2010. As a result, information about the majority of father absence status during three years before 2010 could be imputed for 1,367 children. We used the `predict` function, to which we provided background variables (sex, region of residence, household income, and father absence status in the 1st year of middle school) as new data set for imputation.

#### 4.1.4 Why imputation was not possible for some children and its consequences

Our imputation strategy required that ages and years preceding the reported onset of puberty are covered *within* the age and year range representing the KCYPS sample. Consequently, girls from the oldest cohort who had menarche at younger age could not be imputed for their pre-pubertal father absence status.

Excluding these ‘early starters’ did not bias the distribution of age at menarche (see table below), mainly because there were enough early-starting girls from younger cohorts.

| | Age (years) at menarche | | | |
|---|---|---|---|---|
| | 1st quartile | Median | Mean | 3rd quartile |
| Sample excluding early starters from the oldest cohort (= final analytic sample) | 12.08 | 12.67 | 12.76 | 13.42 |
| Sample including early starters from the oldest cohort | 12.17 | 12.75 | 12.74 | 13.33 |

## 4.2 Imputing household income

To impute household income for at least the three years preceding the beginning of the survey (2007, 2008, 2009), we first constructed a model for log-transformed household income using all the available data on household income from the KCYPS. To reflect the data structure of repeated income observations within individual households, we estimated a mixed-effects model (aka multi-level model), in which fixed-effects were membership to three cohorts, year of survey, mother's highest education, father's highest education, and random-effects were individual ID and region to capture heterogeneity across households and regions. We specified a model as:

$$y_i = \alpha_{j[i]} + \alpha_{k[i]} + X_i\beta + \epsilon_i$$

$$\alpha_j \sim \mathcal{N}(\mu_\alpha, \sigma_\alpha^2), \quad j = 1, \ldots, J$$

$$\alpha_k \sim \mathcal{N}(\mu_\alpha, \sigma_\alpha^2), \quad k = 1, \ldots, K$$

where we have observations of income $y_i$ of $i = 1, \ldots, n$ clustered within individuals $j = 1, \ldots, J$, and within regions $k = 1, \ldots, K$. $\alpha_j$ and $\alpha_j$ are individual intercepts (random-effects) assumed to follow normal distribution. $X_i\beta$ is a matrix of fixed-effects specific to income (membership to three cohorts, year of survey, mother's highest education, father's highest education). According to model comparison, interaction between cohort membership and year of survey improved the model fit. This suggests that, household income not only increased over time, but the degree of increase differed between cohorts. We then used the obtained model, to impute household income for the children whose father absence ($n = 1,367$) we could successfully impute in the abovementioned procedure. Some of them ($n = 23$) had no information on parents' education during the period of interest, and thus could not be imputed for their household income (see Figure 3 in the main text).

## 5 Model outputs for the main results

In this section, we present detailed information on outputs from the Cox proportional-hazard (PH) models (Cox, 1972) we estimated, for the 1) results presented in the main text, and 2) robustness checks we performed by excluding certain subgroups of children from analytic sample (see Section 6 of this document). We conducted all analyses separately by sex.

Our general approach to modeling was as follows. First, we examined if the onset of puberty differed by whether a child did not reside with his biological father during most of the time preceding the event (puberty or censoring). Covariate $X^F$ takes two values: 1 if father is absent, 0 if father is present most of the time before pubertal changes.

$$\lambda(t|X^F) = \lambda_0(t) \cdot \exp(\beta^F X^F), \quad (1)$$

Second, we estimated a Cox proportional hazard model with income category ($X^{In}$ with values "1"="low income", "2"="middle class", "3"="rich") and cohort (categorical $X^C$ with values "1", "2", "3" – corresponding cohort numbers) as additional covariates.

$$\lambda(t|X^F, X^{In}, X^C) = \lambda_0(t) \cdot \exp(\beta^F \cdot X^F + \beta^{In} \cdot X^{In} + \beta^C \cdot X^C), \quad (2)$$

Letting some of $\beta$ equal to zero allowed us to consider different specifications of a model (2), which we compared using Akaike Information Criterion (AIC).

Third, we also checked whether the proportionality assumption holds for used covariates. As we are mainly interested in the effect of father absence, we examined the scaled Schoenfeld residuals for this covariate to look at the dependence between residuals and time. After consideration of results we adopted the extended Cox PH model with time-varying coefficient (Hofner et al., 2011) for the father absence:

$$\lambda(t|X^F, X^{In}, X^C) = \lambda_0(t) \cdot \exp(\beta^F(t) \cdot X^F + \beta^{In} \cdot X^{In} + \beta^C \cdot X^C), \quad (3)$$

where $\beta^F(t) = \beta^F_0 + \beta^F_1 \cdot t$ — a linear function of time.

### 5.1 Boys

Our sample ($n = 3,237$) include all boys with known age at first nocturnal ejaculation or censoring. Hereafter, we refer to the onset of puberty if a boy reported to have experienced the first nocturnal ejaculation.

First, according to likelihood ratio test, there is no evidence that the onset of puberty differs by father absence status (i.e., model (1), Likelihood ratio test 0.7 on 1 df, $p = 0.4$). Second, results from the estimated Cox PH model (2) suggest that cohort, income category and father absence influence the onset of puberty, with different degree of statistical significance.

```
coxph(formula = Surv(bc_age, bc_type) ~ as.factor(cohort) + as.factor(avg_inc_cat) +
fp, data = bck_fpinc_before[cond, ])
n= 3237, number of events= 2034
```

| | coef | exp(coef) | se(coef) | z | Pr(> \|z\|) |
|---|---|---|---|---|---|
| as.factor(cohort)2 | 0.19094 | 1.21038 | 0.06781 | 2.816 | 0.00486 ** |
| as.factor(cohort)3 | 0.36538 | 1.44106 | 0.06664 | 5.483 | 4.19e-08 *** |
| as.factor(avg_inc_cat)2 | 0.40184 | 1.49457 | 0.19814 | 2.028 | 0.04256 * |
| as.factor(avg_inc_cat)3 | 0.52234 | 1.68596 | 0.19910 | 2.623 | 0.00870 ** |
| fp | 0.19369 | 1.21372 | 0.10717 | 1.807 | 0.07073 · |

Signif. codes: *** 0.001, ** 0.01, * 0.05, · 0.1

| | exp(coef) | exp(-coef) | lower .95 | upper .95 |
|---|---|---|---|---|
| as.factor(cohort)2 | 1.210 | 0.8262 | 1.0598 | 1.382 |
| as.factor(cohort)3 | 1.441 | 0.6939 | 1.2646 | 1.642 |
| as.factor(avg_inc_cat)2 | 1.495 | 0.6691 | 1.0136 | 2.204 |
| as.factor(avg_inc_cat)3 | 1.686 | 0.5931 | 1.1412 | 2.491 |
| fp | 1.214 | 0.8239 | 0.9838 | 1.497 |

```
Concordance= 0.539 (se = 0.007 )
Likelihood ratio test= 46.45 on 5 df, p=7e-09
Wald test = 45.48 on 5 df, p=1e-08
Score (logrank) test = 45.74 on 5 df, p=1e-08
```

Third, when we tested for the proportionality assumption, the assumption does not hold for some variables as could be seen form the Table 4.

Table 4: Proportionality assumption test for Cox PH model estimated for boys.

| | $\chi^2$ | df | $p$ |
|---|---|---|---|
| cohort | 48.59 | 2 | 2.8e-11 |
| income | 4.84 | 2 | 0.08875 |
| father absence | 12.56 | 1 | 0.00039 |

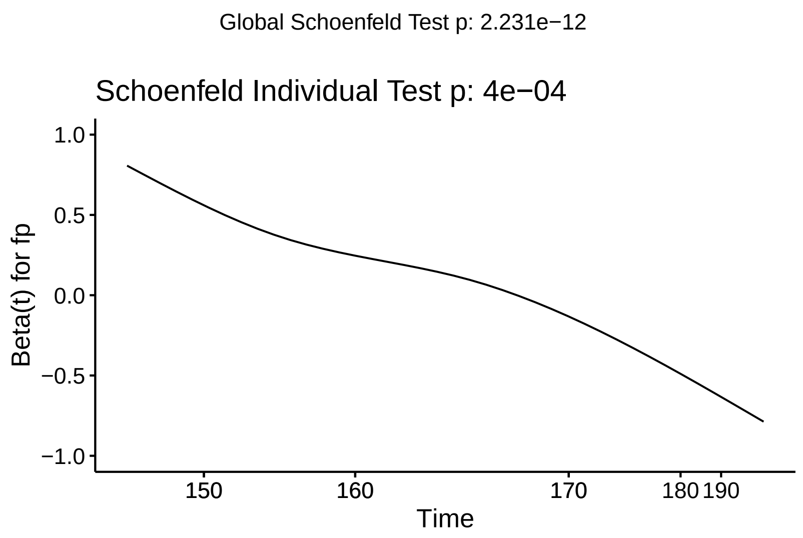


After confirming that the relative hazard of father absence is not constant over time, we re-estimated father absence with a time-varying coefficient as a linear function. We considered two different model specifications, one only with father absence covariate and the other additionally with income category and cohort.

Model (3) with $\beta^F = \beta^F(t)$, $\beta^{In} = 0$ and $\beta^C = 0$:

```
coxph(formula = Surv(bc_age - 107, bc_type) ~ fp + tt(fp), data = bck_fpinc_before[cond, ],
tt = function(x, t, ...) x * t)
n= 3237, number of events= 2034
```

| | coef | exp(coef) | se(coef) | $z$ | Pr(> $\lvert z \rvert$) |
|---|---|---|---|---|---|
| fp | 1.50658 | 4.51128 | 0.39237 | 3.840 | 0.000123 *** |
| tt(fp) | -0.02687 | 0.97349 | 0.00746 | -3.602 | 0.000315 *** |

Signif. codes: *** 0.001, ** 0.01, * 0.05, · 0.1

| | exp(coef) | exp(-coef) | lower .95 | upper .95 |
|---|---|---|---|---|
| fp | 4.5113 | 0.2217 | 2.0908 | 9.7339 |
| tt(fp) | 0.9735 | 1.0272 | 0.9594 | 0.9878 |

```
Concordance= 0.51 (se = 0.003 )
Likelihood ratio test= 13.76 on 2 df, p=0.001
Wald test = 14.94 on 2 df, p=6e-04
Score (logrank) test = 14.64 on 2 df, p=7e-04
```

Model (3) with $\beta^F = \beta^F(t)$, $\beta^{In} \neq 0$ and $\beta^C \neq 0$:

```
coxph(formula = Surv(bc_age - 107, bc_type) ~ as.factor(cohort) +
as.factor(avg_inc_cat) +
fp + tt(fp), data = bck_fpinc_before[cond, ], tt = function(x, t, ...) x * t)
n= 3237, number of events= 2034
```

Table 5: Parameters of Cox PH model with time varying coefficient for father absence estimated for boys sample.

| | coef | se(coef) | exp(coef) | lower .95 | upper .95 | $z$ | Pr(> $\|z\|$) |
|---|---|---|---|---|---|---|---|
| $\beta^{C=2}$ | 0.185929 | 0.067836 | 1.204337 | 1.0544 | 1.3756 | 2.741 | 0.006128 ** |
| $\beta^{C=3}$ | 0.359085 | 0.066677 | 1.432018 | 1.2566 | 1.6319 | 5.385 | 7.23e-08 *** |
| $\beta^{In=2}$ | 0.348277 | 0.197995 | 1.416624 | 0.9610 | 2.0883 | 1.759 | 0.078574 · |
| $\beta^{In=3}$ | 0.465225 | 0.198723 | 1.592372 | 1.0787 | 2.3507 | 2.341 | 0.019228 * |
| $\beta^F_0$ | 1.460607 | 0.397390 | 4.308572 | 1.9773 | 9.3884 | 3.675 | 0.000237 *** |
| $\beta^F_1$ | -0.024239 | 0.007576 | 0.976053 | 0.9617 | 0.9907 | -3.199 | 0.001377 ** |

Signif. codes: *** 0.001, ** 0.01, * 0.05, · 0.1

```
Concordance= 0.545 (se = 0.007 )
Likelihood ratio test= 56.74 on 6 df, p=2e-10
Wald test = 56.93 on 6 df, p=2e-10
Score (logrank) test = 56.85 on 6 df, p=2e-10
```

Table 6: Comparison of different specifications of proportional hazard models fitted to the boys subgroup.

| model specification | covariates | df | AIC |
|---|---|---|---|
| model (1) | father absence | 1 | 29920.42 |
| model (2), $\beta^C = 0$ | income+father absence | 3 | 29912.29 |
| model (2) | cohort+income+father absence | 5 | 29882.67 |
| model (3), $\beta^{In} = 0$, $\beta^C = 0$ | father absence with time varying coef. | 2 | 29909.35 |
| model (3), $\beta^C = 0$ | income+father absence with time varying coef. | 4 | 29902.93 |
| model (3) | cohort+income+father absence with time varying coef. | 6 | 29874.38 |

## 5.2 Girls

For girls, we applied the same procedure taken above. Our sample ($n = 2,861$) include all girls with information on age at pubertal change (including censored cases) measured as menarche.

First, the Cox PH model (1) with just one covariate (father absence) fits the data poorly (Likelihood ratio test = 2.83 on 1 df, $p = 0.09$). Second, if we add cohort and income as covariates (model (2)) we get a better fit. Father absence and income category appear to have little relationship to pubertal timing of girls, according to estimated parameters (see code output below).

```
coxph(formula = Surv(bc_age, bc_type) ~ as.factor(cohort) + as.factor(avg_inc_cat) +
fp, data = bck_fpinc_before[cond, ])
n= 2861, number of events= 2729
```

| | coef | exp(coef) | se(coef) | $z$ | Pr(> $\|z\|$) |
|---|---|---|---|---|---|
| as.factor(cohort)2 | -0.18531 | 0.83085 | 0.04739 | -3.911 | 9.21e-05 *** |
| as.factor(cohort)3 | -0.46956 | 0.62528 | 0.04986 | -9.417 | < 2e-16 *** |
| as.factor(avg_inc_cat)2 | 0.11013 | 1.11642 | 0.17285 | 0.637 | 0.524 |
| as.factor(avg_inc_cat)3 | 0.18645 | 1.20496 | 0.17389 | 1.072 | 0.284 |
| fp | -0.05361 | 0.94780 | 0.09185 | -0.584 | 0.559 |

Signif. codes: *** 0.001, ** 0.01, * 0.05, · 0.1

| | exp(coef) | exp(-coef) | lower .95 | upper .95 |
|---|---|---|---|---|
| as.factor(`cohort`)2 | 0.8308 | 1.2036 | 0.7572 | 0.9117 |
| as.factor(`cohort`)3 | 0.6253 | 1.5993 | 0.5671 | 0.6895 |
| as.factor(`avg_inc_cat`)2 | 1.1164 | 0.8957 | 0.7956 | 1.5666 |
| as.factor(`avg_inc_cat`)3 | 1.2050 | 0.8299 | 0.8569 | 1.6943 |
| `fp` | 0.9478 | 1.0551 | 0.7917 | 1.1347 |

```
Concordance= 0.57 (se = 0.006 )
Likelihood ratio test= 97.53 on 5 df, p=<2e-16
Wald test = 95.96 on 5 df, p=<2e-16
Score (logrank) test = 97.09 on 5 df, p=<2e-16
```

Third, but again as in boys, the proportionality assumption does not hold for all considered variables.

Table 7: Proportionality assumption test for Cox PH model estimated for girls.

| | $\chi^2$ | df | $p$ |
|---|---|---|---|
| cohort | 53.33 | 2 | 2.6e-12 |
| income | 8.46 | 2 | 0.015 |
| father absence | 3.66 | 1 | 0.056 |

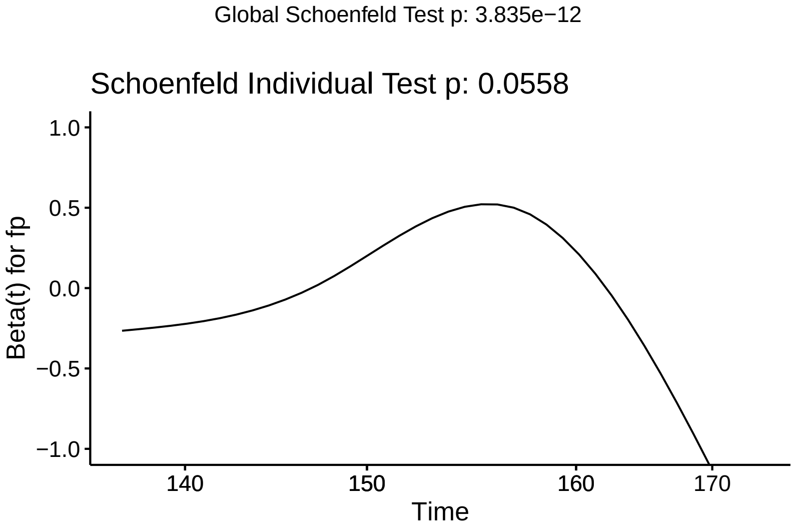


We analyzed the scaled Schoenfeld residuals for father absence covariate to check at the dependence between residuals and time. They form a pattern which is far from horizontal line. It is an exaggeration to assume a linear decline of $\beta(t)$ with time, but for the sake of comparability with analysis conducted for boys, and since we do not actually know the mechanisms behind the effect of father absence changing with age in such a fancy "waving" way, we stick to the simplest model and assume that the effect of father absence changes linearly with age.

The model output from R for two different model specifications is given below.
Model (3) with $\beta^F = \beta^F(t)$, $\beta^{In} = 0$ and $\beta^C = 0$:

```
coxph(formula = Surv(bc_age - 107, bc_type) ~ fp + tt(fp),
data = bck_fpinc_before[cond, ], tt = function(x, t, ...) x * t)
n= 2861, number of events= 2729
```

| | coef | exp(coef) | se(coef) | $z$ | Pr(> $\|z\|$) |
|---|---|---|---|---|---|
| `fp` | 0.473020 | 1.604833 | 0.307936 | 1.536 | 0.125 |
| `tt(fp)` | -0.013162 | 0.986924 | 0.006473 | -2.033 | 0.042 * |

Signif. codes: *** 0.001, ** 0.01, * 0.05, · 0.1

| | exp(coef) | exp(-coef) | lower .95 | upper .95 |
|---|---|---|---|---|
| fp | 1.6048 | 0.6231 | 0.8776 | 2.9346 |
| tt(fp) | 0.9869 | 1.0132 | 0.9745 | 0.9995 |

```
Concordance= 0.499 (se = 0.003 )
Likelihood ratio test= 6.98 on 2 df, p=0.03
Wald test = 6.47 on 2 df, p=0.04
Score (logrank) test = 6.53 on 2 df, p=0.04
```

Model (3) with $\beta^F = \beta^F(t)$, $\beta^{In} \neq 0$ and $\beta^C \neq 0$:

```
coxph(formula = Surv(bc_age - 107, bc_type) ~ as.factor(cohort) +
as.factor(avg_inc_cat) + fp + tt(fp),
data = bck_fpinc_before[cond,], tt = function(x, t, ...) x * t)
n= 2861, number of events= 2729
```

Table 8: Parameters of Cox PH model with time varying coefficient for father absence estimated for girls sample.

| | coef | se(coef) | exp(coef) | lower .95 | upper .95 | $z$ | Pr(> $\|z\|$) |
|---|---|---|---|---|---|---|---|
| $\beta^{C=2}$ | -0.186032 | 0.047381 | 0.830247 | 0.7566 | 0.9110 | -3.926 | 8.63e-05 *** |
| $\beta^{C=3}$ | -0.471471 | 0.049880 | 0.624084 | 0.5660 | 0.6882 | -9.452 | < 2e-16 *** |
| $\beta^{In=2}$ | 0.089514 | 0.172771 | 1.093642 | 0.7795 | 1.5344 | 0.518 | 0.6044 |
| $\beta^{In=3}$ | 0.166014 | 0.173737 | 1.180589 | 0.8399 | 1.6595 | 0.956 | 0.3393 |
| $\beta^F_0$ | 0.594009 | 0.307422 | 1.811235 | 0.9915 | 3.3087 | 1.932 | 0.0533 · |
| $\beta^F_1$ | -0.013959 | 0.006448 | 0.986138 | 0.9738 | 0.9987 | -2.165 | 0.0304 * |

Signif. codes: *** 0.001, ** 0.01, * 0.05, · 0.1

```
Concordance= 0.569 (se = 0.006 )
Likelihood ratio test= 102.2 on 6 df, p=<2e-16
Wald test = 100.2 on 6 df, p=<2e-16
Score (logrank) test = 101.4 on 6 df, p=<2e-16
```

Table 9: Comparison of different specifications of proportional hazard models fitted to the girls subgroup.

| model specification | covariates | df | AIC |
|---|---|---|---|
| model (1) | father absence | 1 | 38184.31 |
| model (2), $\beta^C = 0$ | income+father absence | 3 | 38185.98 |
| model (2) | cohort+income+father absence | 5 | 38097.61 |
| model (3), $\beta^{In} = 0$, $\beta^C = 0$ | father absence with time varying coef. | 2 | 38182.17 |
| model (3), $\beta^C = 0$ | income+father absence with time varying coef. | 4 | 38183.96 |
| model (3) | cohort+income+father absence with time varying coef. | 6 | 38094.92 |

### 5.3 Conclusion

In boys, extended Cox PH model (3) with time-varying coefficient fits the data better than the Cox PH model with constant coefficient (2), even if we consider just one covariate (Table 6). We therefore chose to base our main results on the "full model" shown at the bottom of Table 6, that is, the Cox PH model (3) with $\beta^F = \beta^F(t)$, $\beta^{In} \neq 0$ and $\beta^C \neq 0$, the outputs of which are shown above (Section 5.1) and estimated paremeters are presented in a Table 5.

In girls, as in boys, we find that the "full" model with all three covariates and time-dependent coefficient for father absence gives the best fit (model specification at the bottom of Table 9). We therefore base our main results on this model. The outputs of it are shown above (Section 5.2) and estimated parameters of this model are presented in the Table 8.

# 6 Sensitivity analyses

## 6.1 Excluding the imputed sample

The model which we selected for the analysis was applied to the sample without imputed information (boys – 3073, girls – 2496). For those who started puberty in 2010 we used information on father absence and income before the onset of puberty for the same year.

Below we present some R output of model (3) for different sexes and model comparison statistics, considering cohort as a covariate.

### Boys

```
coxph(formula = Surv(bc_age - 107, bc_type) ~ as.factor(cohort) +
as.factor(avg_inc_cat) + fp + tt(fp), data =bck_fpinc_before[cond,],
tt = function(x, t, ...) x * t)
n= 3073, number of events= 1870
```

| | coef | exp(coef) | se(coef) | $z$ | $\Pr(>\lvert z\rvert)$ |
|---|---|---|---|---|---|
| as.factor(cohort)2 | 0.109743 | 1.115991 | 0.068498 | 1.602 | 0.10912 |
| as.factor(cohort)3 | 0.111999 | 1.118511 | 0.069332 | 1.615 | 0.10622 |
| as.factor(avg_inc_cat)2 | 0.283985 | 1.328413 | 0.199022 | 1.427 | 0.15361 |
| as.factor(avg_inc_cat)3 | 0.362271 | 1.436588 | 0.199812 | 1.813 | 0.06982 · |
| fp | 1.155440 | 3.175419 | 0.442409 | 2.612 | 0.00901 ** |
| tt(fp) | -0.018993 | 0.981186 | 0.008121 | -2.339 | 0.01935 * |

Signif. codes: *** 0.001, ** 0.01, * 0.05, · 0.1

| | exp(coef) | exp(-coef) | lower .95 | upper .95 |
|---|---|---|---|---|
| as.factor(cohort)2 | 1.1160 | 0.8961 | 0.9758 | 1.2763 |
| as.factor(cohort)3 | 1.1185 | 0.8940 | 0.9764 | 1.2813 |
| as.factor(avg_inc_cat)2 | 1.3284 | 0.7528 | 0.8993 | 1.9622 |
| as.factor(avg_inc_cat)3 | 1.4366 | 0.6961 | 0.9711 | 2.1253 |
| fp | 3.1754 | 0.3149 | 1.3342 | 7.5575 |
| tt(fp) | 0.9812 | 1.0192 | 0.9657 | 0.9969 |

```
Concordance= 0.516  (se = 0.007 )
Likelihood ratio test= 14.92  on 6 df,   p=0.02
Wald test            = 14.8  on 6 df,   p=0.02
Score (logrank) test = 14.7  on 6 df,   p=0.02
```

Model comparison:

| model specification | covariates | df | AIC |
|---|---|---|---|
| model (1) | father absence | 1 | 27278.59 |
| model (2), $\beta^C = 0$ | income+father absence | 3 | 27276.57 |
| model (2) | cohort+income+father absence | 5 | 27277.23 |
| model (3), $\beta^{In} = 0$, $\beta^C = 0$ | father absence with time varying coef. | 2 | 27273.92 |
| model (3), $\beta^C = 0$ | income+father absence with time varying coef. | 4 | 27272.81 |
| model (3) | cohort+income+father absence with time varying coef. | 6 | 27273.75 |

### Girls

```
coxph(formula = Surv(bc_age - 107, bc_type) ~ as.factor(cohort) +
as.factor(avg_inc_cat) + fp + tt(fp), data = bck_fpinc_before[cond, ],
tt = function(x, t, ...) x * t)
n= 2496, number of events= 2364
```

| | coef | exp(coef) | se(coef) | $z$ | Pr(> $|z|$) |
|---|---|---|---|---|---|
| as.factor(cohort)2 | -0.229856 | 0.794648 | 0.047795 | -4.809 | 1.52e-06 *** |
| as.factor(cohort)3 | -0.933600 | 0.393136 | 0.059375 | -15.724 | < 2e-16 *** |
| as.factor(avg_inc_cat)2 | 0.074861 | 1.077734 | 0.175983 | 0.425 | 0.6706 |
| as.factor(avg_inc_cat)3 | 0.097790 | 1.102731 | 0.177120 | 0.552 | 0.5809 |
| fp | 0.695845 | 2.005403 | 0.316871 | 2.196 | 0.0281 * |
| tt(fp) | -0.016579 | 0.983558 | 0.006535 | -2.537 | 0.0112 * |

Signif. codes: *** 0.001, ** 0.01, * 0.05, · 0.1

| | exp(coef) | exp(-coef) | lower .95 | upper .95 |
|---|---|---|---|---|
| as.factor(cohort)2 | 0.7946 | 1.2584 | 0.7236 | 0.8727 |
| as.factor(cohort)3 | 0.3931 | 2.5437 | 0.3499 | 0.4417 |
| as.factor(avg_inc_cat)2 | 1.0777 | 0.9279 | 0.7633 | 1.5216 |
| as.factor(avg_inc_cat)3 | 1.1027 | 0.9068 | 0.7793 | 1.5604 |
| fp | 2.0054 | 0.4987 | 1.0777 | 3.7318 |
| tt(fp) | 0.9836 | 1.0167 | 0.9710 | 0.9962 |

```
Concordance= 0.614  (se = 0.005 )
Likelihood ratio test= 295.2  on 6 df,   p=<2e-16
Wald test            = 264.3  on 6 df,   p=<2e-16
Score (logrank) test = 276.8  on 6 df,   p=<2e-16
```

Model comparison:

| model specification | covariates | df | AIC |
|---|---|---|---|
| model (1) | father absence | 1 | 32423.09 |
| model (2), $\beta^C = 0$ | income+father absence | 3 | 32426.92 |
| model (2) | cohort+income+father absence | 5 | 32145.32 |
| model (3), $\beta^{In} = 0$, $\beta^C = 0$ | father absence with time varying coef. | 2 | 32420.39 |
| model (3), $\beta^C = 0$ | income+father absence with time varying coef. | 4 | 32424.22 |
| model (3) | cohort+income+father absence with time varying coef. | 6 | 32140.92 |

Conclusion on robustness check We find that, overall, the estimated survival patterns stayed the same after excluding the sample for which father absence had to be imputed. Even in reduced sample, the influence of father absence on the onset of puberty is time-dependent and more pronounced in boys. For girls, model (3) with cohort and income covariates fits the data better than the others. For boys, cohort as covariate does not improves fit but increases the number of degrees of freedom.

### 6.2 Excluding the oldest cohort

The model which we selected for the analysis was applied to the sample without data for the oldest cohort (2180 boys, 2001 girls) but including imputed data. Below we present some R output of model (3) for different sexes.

Boys

```
coxph(formula = Surv(bc_age - 107, bc_type) ~ as.factor(cohort) +
    as.factor(avg_inc_cat) + fp + tt(fp), data = dt_m
    , tt = function(x,t, ...) x * t)
  n= 2180, number of events= 1121
                            coef exp(coef) se(coef)      z Pr(>|z|)
as.factor(cohort)2        0.25167   1.28617  0.06946  3.623 0.000291 ***
as.factor(avg_inc_cat)2   0.05623   1.05784  0.24871  0.226 0.821140
as.factor(avg_inc_cat)3   0.04320   1.04415  0.24983  0.173 0.862721
fp                        1.59617   4.93409  0.57800  2.762 0.005753 **
tt(fp)                   -0.03127   0.96922  0.01174 -2.663 0.007747 **
---
Signif. codes:  0 ‘***’ 0.001 ‘**’ 0.01 ‘*’ 0.05 ‘.’ 0.1 ‘ ’ 1

                         exp(coef) exp(-coef) lower .95 upper .95
as.factor(cohort)2          1.2862     0.7775    1.1225    1.4738
as.factor(avg_inc_cat)2     1.0578     0.9453    0.6497    1.7224
as.factor(avg_inc_cat)3     1.0441     0.9577    0.6399    1.7038
fp                          4.9341     0.2027    1.5893   15.3179
tt(fp)                      0.9692     1.0318    0.9472    0.9918

Concordance= 0.547  (se = 0.009 )
Likelihood ratio test= 20.99  on 5 df,   p=8e-04
Wald test            = 21.56  on 5 df,   p=6e-04
Score (logrank) test = 21.49  on 5 df,   p=7e-04
```

Girls

```
coxph(formula = Surv(bc_age - 107, bc_type) ~ as.factor(cohort) +
    as.factor(avg_inc_cat) + fp + tt(fp), data = dt_f
    , tt = function(x, t, ...) x * t)
  n= 2001, number of events= 1871
                             coef exp(coef)  se(coef)      z Pr(>|z|)
as.factor(cohort)2       -0.168989  0.844518  0.048003 -3.520 0.000431 ***
as.factor(avg_inc_cat)2   0.009464  1.009509  0.183565  0.052 0.958880
as.factor(avg_inc_cat)3  -0.033597  0.966961  0.184222 -0.182 0.855290
fp                        0.533716  1.705257  0.379743  1.405 0.159883
tt(fp)                   -0.014647  0.985459  0.008470 -1.729 0.083760 .
---
Signif. codes:  0 ‘***’ 0.001 ‘**’ 0.01 ‘*’ 0.05 ‘.’ 0.1 ‘ ’ 1

                         exp(coef) exp(-coef) lower .95 upper .95
as.factor(cohort)2          0.8445     1.1841    0.7687    0.9278
as.factor(avg_inc_cat)2     1.0095     0.9906    0.7045    1.4466
as.factor(avg_inc_cat)3     0.9670     1.0342    0.6739    1.3875
```

```
fp                            1.7053     0.5864    0.8101    3.5894
tt(fp)                        0.9855     1.0148    0.9692    1.0020

Concordance= 0.521  (se = 0.007 )
Likelihood ratio test= 16.87  on 5 df,   p=0.005
Wald test            = 16.72  on 5 df,   p=0.005
Score (logrank) test = 16.76  on 5 df,   p=0.005
```

Conclusion on robustness check after exclusion of the oldest cohort from the sample. The model detects influence of father absence on the onset of puberty as time-dependent in boys even if the oldest cohort is excluded from the sample. According to proposed model father absence has no significant effect on puberty in girls.

## 6.3 Excluding the cases of partial father absence

Here, we sought to examine the association between father absence and pubertal timing, after excluding cases in which father-present status fluctuated over time (i.e., partial father absence). To simplify the process, we undertook this excercise without the cases for which we had to impute pre-pubertal father-absence status, because imputation requires preparation of modeling data set with accordance to adopted definition of farther absence. For those who started puberty in 2010, used information on father absence and income for the same year. Thus, the results presented here can be best compared directly with another sensitivity analysis on the sample excluding the imputed cases (see section 6.1). We re-ran the same models as for the main analyses with time-varying father absence effect.

Below we present some R output of model (3), separately by sex.

### Boys

```
coxph(formula = Surv(bc_age - 107, bc_type) ~ as.factor(cohort) +
    as.factor(avg_inc_cat) + fp + tt(fp), data = dt_m
    , tt = function(x, t, ...) x * t)
  n= 2967, number of events= 1824
                              coef exp(coef)  se(coef)      z Pr(>|z|)
as.factor(cohort)2        0.121007  1.128633  0.069456  1.742   0.0815 .
as.factor(cohort)3        0.108152  1.114217  0.070389  1.536   0.1244
as.factor(avg_inc_cat)2   0.166465  1.181122  0.204130  0.815   0.4148
as.factor(avg_inc_cat)3   0.221197  1.247569  0.205065  1.079   0.2807
fp                        1.104534  3.017817  0.456870  2.418   0.0156 *
tt(fp)                   -0.015292  0.984824  0.008371 -1.827   0.0677 .
---
Signif. codes:  0 ‘***’ 0.001 ‘**’ 0.01 ‘*’ 0.05 ‘.’ 0.1 ‘ ’ 1

                         exp(coef) exp(-coef) lower .95 upper .95
as.factor(cohort)2          1.1286     0.8860    0.9850     1.293
as.factor(cohort)3          1.1142     0.8975    0.9706     1.279
as.factor(avg_inc_cat)2     1.1811     0.8467    0.7917     1.762
as.factor(avg_inc_cat)3     1.2476     0.8016    0.8347     1.865
fp                          3.0178     0.3314    1.2326     7.389
tt(fp)                      0.9848     1.0154    0.9688     1.001

Concordance= 0.528  (se = 0.008 )
Likelihood ratio test= 13.28  on 6 df,   p=0.04
Wald test            = 14.26  on 6 df,   p=0.03
Score (logrank) test = 14.32  on 6 df,   p=0.03
```

### Girls

```
coxph(formula = Surv(bc_age - 107, bc_type) ~ as.factor(cohort) +
    as.factor(avg_inc_cat) + fp + tt(fp), data = dt_f
    , tt = function(x, t, ...) x * t)
  n= 2403, number of events= 2277
                              coef exp(coef)  se(coef)       z Pr(>|z|)
as.factor(cohort)2       -0.221228  0.801534  0.048922  -4.522 6.12e-06 ***
as.factor(cohort)3       -0.923133  0.397272  0.060029 -15.378  < 2e-16 ***
as.factor(avg_inc_cat)2   0.051411  1.052755  0.193982   0.265   0.7910
as.factor(avg_inc_cat)3   0.061881  1.063836  0.194668   0.318   0.7506
```

```
fp                         0.760822  2.140036  0.351535   2.164   0.0304 *
tt(fp)                    -0.016800  0.983340  0.007122  -2.359   0.0183 *
---
Signif. codes:  0 ‘***’ 0.001 ‘**’ 0.01 ‘*’ 0.05 ‘.’ 0.1 ‘ ’ 1

                          exp(coef) exp(-coef) lower .95 upper .95
as.factor(cohort)2           0.8015     1.2476    0.7282    0.8822
as.factor(cohort)3           0.3973     2.5172    0.3532    0.4469
as.factor(avg_inc_cat)2      1.0528     0.9499    0.7198    1.5397
as.factor(avg_inc_cat)3      1.0638     0.9400    0.7264    1.5580
fp                           2.1400     0.4673    1.0745    4.2623
tt(fp)                       0.9833     1.0169    0.9697    0.9972

Concordance= 0.615  (se = 0.005 )
Likelihood ratio test= 283.3  on 6 df,   p=<2e-16
Wald test            = 254.1  on 6 df,   p=<2e-16
Score (logrank) test = 266.1  on 6 df,   p=<2e-16
```

Conclusion on robustness check after excluding the cases of partial father absence. We find that our conclusion about the association between father absence and pubertal timing remains, even after removing cases where biological father was reported to be present and absent in the child's household over time. The estimated magnitude of the effect of father absence is slightly different from the one reported in the section 6.1, but still shows that the impact is larger and significant only in boys.

## 6.4 Averaging inconsistent answers

Here, we examined if our findings differ when inconsistent answers about the beginning of puberty are handled differently (taking averages). Same as the sensitivity analysis conducted for the sample excluding the cases of partial father absence (Section 6.3), we undertook this excercise without the cases for which we had to impute pre-pubertal father-absence status which requires additional preparation of modeling data set coherent with definitions assumed. For those who started puberty in 2010 we used information on father absence and income for the same year. Thus, the results presented here can be best compared directly with another sensitivity analysis on the sample excluding the imputed cases (see section 6.1). We re-ran the same models as for the main analyses with time-varying father absence effect.

Below we present some R outputs of the model (3), separately by sex.

### Boys

```
Call:
coxph(formula = Surv(bc_age - 107, bc_type) ~ as.factor(cohort) +
    as.factor(avg_inc_cat) + fp + tt(fp), data = dt_m
    , tt = function(x,t, ...) x * t)
  n= 3042, number of events= 1839
                             coef exp(coef)  se(coef)      z Pr(>|z|)
as.factor(cohort)2      -0.057248  0.944360  0.069759 -0.821   0.4118
as.factor(cohort)3      -0.151655  0.859285  0.071292 -2.127   0.0334 *
as.factor(avg_inc_cat)2  0.327830  1.387953  0.202340  1.620   0.1052
as.factor(avg_inc_cat)3  0.380074  1.462393  0.203292  1.870   0.0615 .
fp                       0.948229  2.581135  0.463136  2.047   0.0406 *
tt(fp)                  -0.015289  0.984827  0.008181 -1.869   0.0616 .
---
Signif. codes:  0 ‘***’ 0.001 ‘**’ 0.01 ‘*’ 0.05 ‘.’ 0.1 ‘ ’ 1

                        exp(coef) exp(-coef) lower .95 upper .95
as.factor(cohort)2         0.9444     1.0589    0.8237    1.0827
as.factor(cohort)3         0.8593     1.1638    0.7472    0.9881
as.factor(avg_inc_cat)2    1.3880     0.7205    0.9336    2.0635
as.factor(avg_inc_cat)3    1.4624     0.6838    0.9818    2.1782
fp                         2.5811     0.3874    1.0413    6.3978
tt(fp)                     0.9848     1.0154    0.9692    1.0007

Concordance= 0.537  (se = 0.007 )
Likelihood ratio test= 13.65  on 6 df,   p=0.03
Wald test            = 13.19  on 6 df,   p=0.04
Score (logrank) test = 13.17  on 6 df,   p=0.04
```

### Girls

```
coxph(formula = Surv(bc_age - 107, bc_type) ~ as.factor(cohort) +
    as.factor(avg_inc_cat) + fp + tt(fp), data = dt_f
    , tt = function(x,  t, ...) x * t)
  n= 2417, number of events= 2285
                             coef exp(coef)  se(coef)       z Pr(>|z|)
as.factor(cohort)2      -0.249058  0.779535  0.048013  -5.187 2.13e-07 ***
as.factor(cohort)3      -1.075346  0.341180  0.063792 -16.857  < 2e-16 ***
as.factor(avg_inc_cat)2  0.034939  1.035556  0.176200   0.198  0.84282
```

```
as.factor(avg_inc_cat)3   0.062209  1.064184  0.177316   0.351  0.72571
fp                        0.686608  1.986964  0.316865   2.167  0.03024 *
tt(fp)                   -0.016683  0.983456  0.006475  -2.576  0.00998 **
---
Signif. codes:  0 ‘***’ 0.001 ‘**’ 0.01 ‘*’ 0.05 ‘.’ 0.1 ‘ ’ 1

                         exp(coef) exp(-coef) lower .95 upper .95
as.factor(cohort)2          0.7795     1.2828    0.7095    0.8565
as.factor(cohort)3          0.3412     2.9310    0.3011    0.3866
as.factor(avg_inc_cat)2     1.0356     0.9657    0.7331    1.4627
as.factor(avg_inc_cat)3     1.0642     0.9397    0.7518    1.5064
fp                          1.9870     0.5033    1.0678    3.6975
tt(fp)                      0.9835     1.0168    0.9711    0.9960

Concordance= 0.614  (se = 0.006 )
Likelihood ratio test= 347  on 6 df,   p=<2e-16
Wald test            = 300.9  on 6 df,   p=<2e-16
Score (logrank) test = 318.8  on 6 df,   p=<2e-16
```

Conclusion on the robustness checks when inconsistent answers are averaged We find that averaging the inconsistent reports of pubertal age still gives the estimated survival patterns align with the main findings. The influence of father absence on the onset of puberty is time-dependent and more pronounced in boys.

## Bibliography


Bauer, P. J. and M. Larkina (2016). Predicting remembering and forgetting of autobiographical memories in children and adults: A 4-year prospective study. *Memory 24*(10), 1345–1368.

Cox, D. R. (1972). Regression models and life-tables. *Journal of the Royal Statistical Society. Series B (Methodological) 34*(2), 187–220.

Hofner, B., T. Kneib, W. Hartl, and H. Küchenhoff (2011). Building cox-type structured hazard regression models with time-varying effects. *Statistical Modelling 11*(1), 3–24.
